# Muscle Quantity at C3 and/or L3 on Routine Trauma Series Computed Tomography Correlate With Brain Frailty and Clinical Frailty Scale: A Cross-Sectional Study

**DOI:** 10.7759/cureus.15912

**Published:** 2021-06-25

**Authors:** Austin R Gomindes, Jason P Appleton, Ruchi Chugh, Carly Welch

**Affiliations:** 1 Trauma and Orthopaedics, Queen Elizabeth Hospital Birmingham, Birmingham, GBR; 2 Medical Education, University of Edinbrugh, Edinbrugh, GBR; 3 Stroke, University Hospital Birmingham, NHS Foundation Trust, Birmingham, GBR; 4 Geriatric Medicine, University Hospitals Birmingham, NHS Foundation Trust, Birmingham, GBR; 5 Medical Research Council (MRC) - Versus Arthritis Centre for Musculoskeletal Ageing Research, University of Birmingham and University of Nottingham, Birmingham, GBR; 6 Institute of Inflammation and Ageing, College of Medical and Dental Sciences, University of Birmingham, Birmingham, GBR

**Keywords:** sarcopenia, clinical frailty, physical frailty, clinical frailty score, brain frailty, brain frailty score

## Abstract

Background

Sarcopenia (low muscle mass and function) is increasingly recognised to impact the quality of life and patient outcomes. The relationship with brain frailty is unknown.

Objectives

Assess if muscle mass at C3 correlates with muscle mass at L3 on routine trauma imaging. Assess for associations between muscle mass, brain frailty, and Clinical Frailty Scale (CFS) on routine trauma imaging.

Methods

Routine trauma-series computed tomography (CT) scans were retrospectively analysed for patients aged ≥16-years-old admitted to Queen Elizabeth Hospital in January 2020. Paravertebral, sternocleidomastoid, and total muscle cross-sectional area (CSA) at C3 (C3-SMM), and total psoas muscle CSA (TPA), total muscle CSA (L3-SMM), and total adipose CSA at L3 were calculated. Brain frailty scores were calculated assessing for leukoaraiosis, cerebral atrophy, and old vascular lesions/infarcts. CFS was calculated retrospectively from clinical notes. We assessed for correlation against age, CFS, muscle mass, and brain frailty using Pearson’s correlations.

Results

We included 111 patients in this study (mean age 49, SD 25.6; 65.8% female). C3-SMM strongly correlated with L3-SMM (r=0.746, p<0.001). Paravertebral and sternocleidomastoid CSA correlated with C3-SMM (paravertebral: r=0.814, p<0.001; sternocleidomastoid: r=0.814, p<0.001). TPA strongly correlated with L3-SMM (r=0.800, p<0.001). Sternocleidomastoid CSA and TPA both negatively correlated moderately with age (sternocleidomastoid: r=−0.460, p<0.001; TPA: r=−0.468, p<0.001), CFS (sternocleidomastoid: r=−0.414, p<0.001; TPA: r=−0.431, p<0.001), and brain frailty (sternocleidomastoid: r=−0.395, p<0.001; TPA: r=−0.436, p<0.001). Adipose CSA at L3 did not correlate with age, CFS, brain frailty, or muscle mass.

Conclusion

Muscle mass at C3 relates to muscle mass at L3. Muscle mass on routine trauma imaging is negatively associated with age, CFS, and brain frailty.

## Introduction

As life expectancy has increased alongside earlier population growth, the proportion of the population living into older age has increased. However, whilst lifespan has increased, the number of expected years spent in healthy living has not increased [1]. Thus, an increasing proportion of patients presenting to emergency departments are older, and with pre-existent health problems [2]. This is especially true for major trauma.

Frailty is a syndrome of heightened vulnerability to reduced likelihood of resolution of homeostasis following a stressor event [3]. The prevalence of frailty increases with age. The Clinical Frailty Scale (CFS) is increasingly utilised to operationalise frailty in clinical practice. Patients with frailty have complex needs, and management of trauma in these patients necessitates a multi-dimensional multi-disciplinary approach. Older patients, particularly with frailty, are more likely to present with more severe injuries from less severe mechanisms. Additionally, injuries may not be accompanied by readily discernible symptoms or signs [2]. Within the United Kingdom, data from the Trauma Audit Research Network has shown that the commonest mechanism of injury amongst older adults is a fall from standing height, with the head and thorax being the commonest body areas injured.

Both cognitive impairment and sarcopenia are known risk factors for falls and injuries amongst older adults. Sarcopenia is defined as reduced muscle strength with reduced muscle quantity or quality [4]. The prevalence of sarcopenia increases with age. It is related to but distinct from frailty. Although imaging does not measure either muscle or cognitive function, it may be used as an adjunct to assessment. Muscle quantity at the level of the third lumbar vertebra (L3) on routine imaging is increasingly recognised as a useful surrogate of total body skeletal muscle mass. More recently, muscle quantity at L3 has been shown to correlate with muscle quantity at the level of the third cervical vertebra (C3) in head and neck cancer patients. Similarly, brain frailty on CT brain scans has been associated with worse clinical outcomes following stroke, but it is unclear whether these imaging markers are associated with clinical frailty or with outcomes in other patient groups.

Objectives of the study

Assess if muscle mass at C3 correlates with muscle mass at L3 on routine trauma imaging and assess for associations between muscle mass, brain frailty, and CFS on routine trauma imaging.

## Materials and methods

Study population

Patients were identified retrospectively utilising routine health informatics systems for clinical quality purposes.

Inclusion Criteria

Patients aged 16 years and older, computed tomography (CT) trauma-series imaging (head to pelvis) performed within the Queen Elizabeth Hospital Birmingham (QEHB) emergency department, University Hospitals Birmingham NHS Foundation Trust (UHBFT), and admitted between January 01, 2020 and January 31, 2020 were included.

Exclusion Criterion

The patient presented to any other emergency department of the University Hospitals of Birmingham was excluded.

Ethical considerations

Data were collected for this study locally as part of a service evaluation for patients undergoing trauma-series imaging. This was approved by the UHBFT clinical governance team (CARMS-16892). No changes were made to patient care and all data were accessed by the clinical team only. Alongside guidance set out by the Health Research Authority [5] review by a national research ethics committee was not required. Approval was obtained for secondary analysis of the anonymised database from the University of Birmingham Science, Technology, Engineering, and Mathematics Ethical Review Committee (ERN-21-0281).

Clinical information

Age, gender, ethnicity, previous residence and care requirements, discharge destination, and length of hospital stay were extracted from routine clinical noting. CFS was calculated retrospectively by a single clinician considering documented functional status and dependency two weeks prior to admission. Death within one year of admission was recorded through clinical records linked to the National Health Service, United Kingdom (NHS) Digital Spine. Trauma CT findings were recorded from prospective clinical reports as binary variables according to the location of the injury. Data were extracted and anonymised by the clinical team. 

Calculation of muscle quantity

Muscle cross-sectional area (CSA) was measured using the CareStream VUE-PACS Client Software Freehand ROI tool (CareStream Health, Inc., Rochester, NY). Image analysis was performed as described in previous reports [6,7].

The third cervical vertebra (C3) was chosen as the reference point in the neck, and the third lumbar vertebra (L3) as the reference point in the abdomen. Image selection was performed using a standard procedure: by scrolling through the C3 or L3 vertebra in a caudate to cephalad direction, the first CT-slide to completely show the entire vertebral arch and the transverse and spinous processes was selected. 

**Figure 1:**
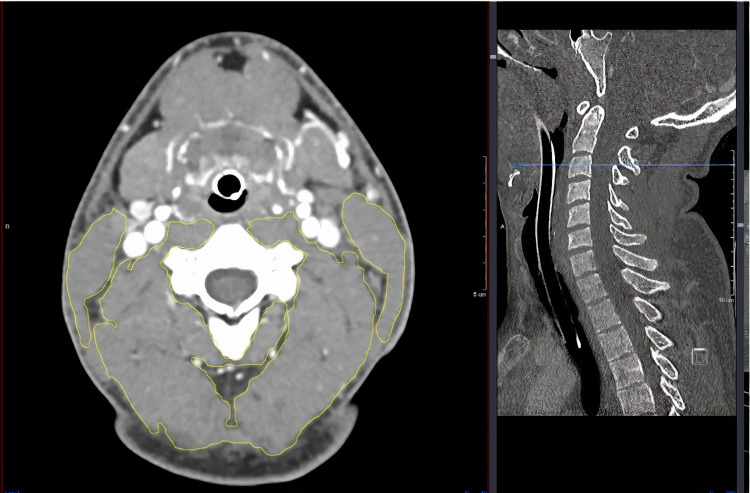
Axial CT image of total skeletal muscle (left) delineated in yellow at the level of the C3 vertebra

**Figure 2:**
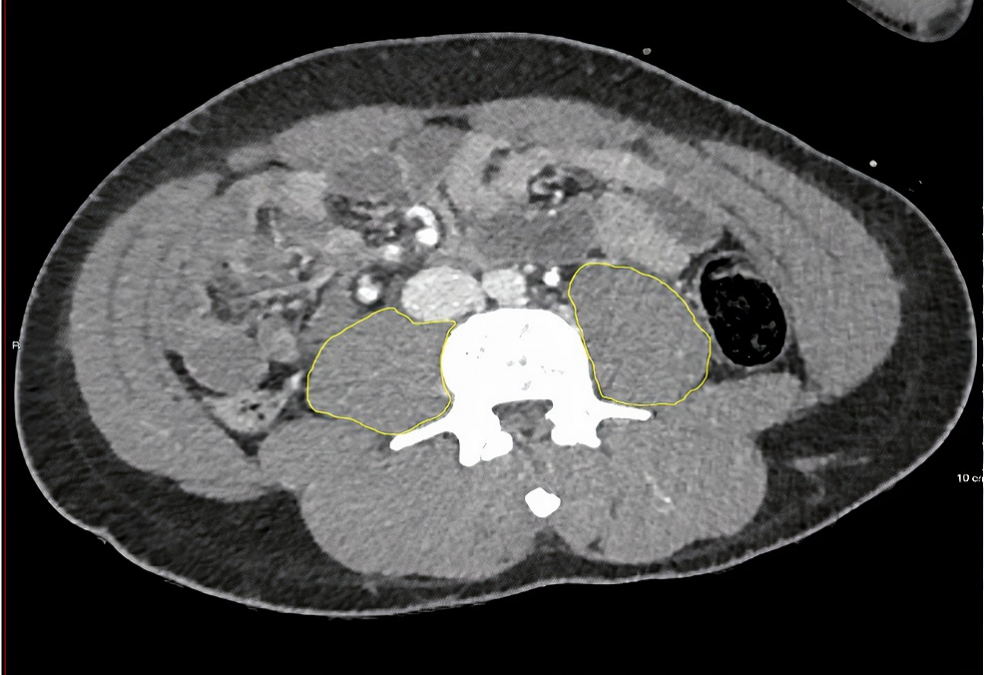
Axial CT image of total psoas area delineated in yellow at the level of the L3 vertebra

**Figure 3:**
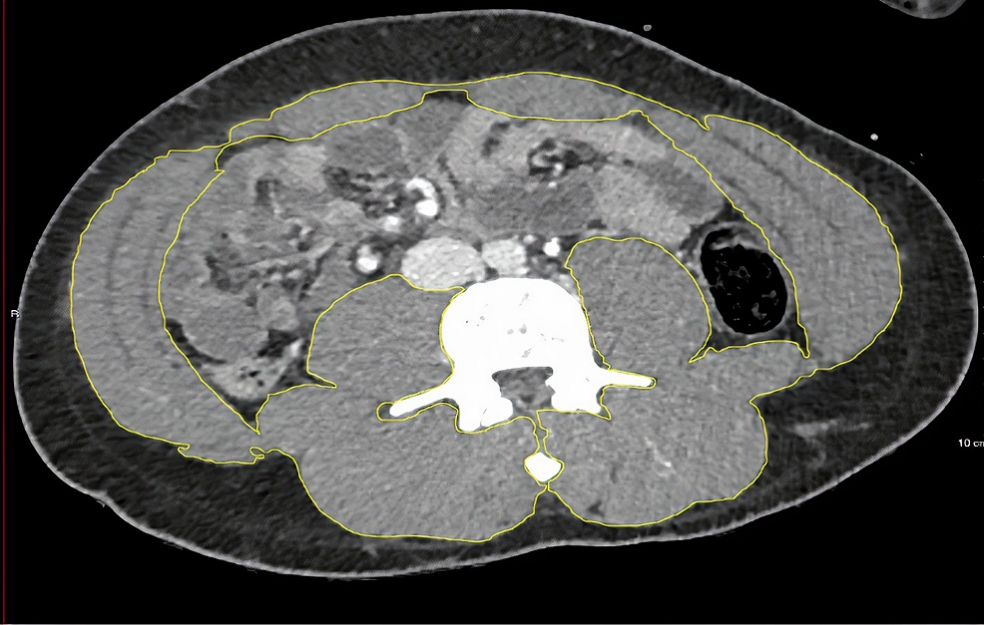
Axial CT image of total skeletal muscle area delineated in yellow at the level of the L3 vertebra

Skeletal muscle was identified using standard Hounsfield unit (HU) ranges, being −29 to +151 HU [8,9]. Delineation of the muscles was performed manually by a single clinician who first delineated muscle quantity at C3 for all patients, and then delineated muscle quantity at L3 for all patients. After delineation, the range given was looked at; if outside the tissue density range (−29 to +151 HU) [6], the measurements were re-taken. Para-vertebral muscle (PVM) and sternocleidomastoid muscle (SCM) CSA were measured separately at C3 (Figure 1). At L3, the total psoas area (TPA; Figure 2) was calculated as the combined right and left psoas CSA. Total skeletal muscle mass (SMM-L3; Figure 3) was calculated by delineating all muscles at L3. Adipose tissue was also measured at L3 with reference tissue density range (HU −90 to −30).

Calculation of brain frailty score

**Figure 4:**
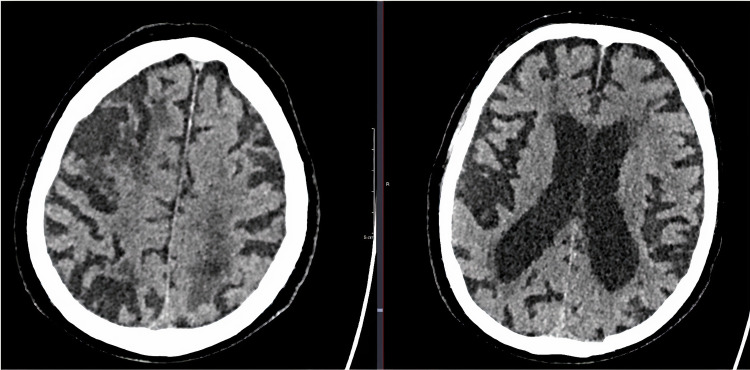
Cerebral atrophy showing cortical cerebral atrophy and central atrophy Cerebral atrophy showing cortical cerebral atrophy identified by the loss of cortical cerebral parenchyma; central cerebral atrophy demonstrated by ventriculomegaly.

**Figure 5:**
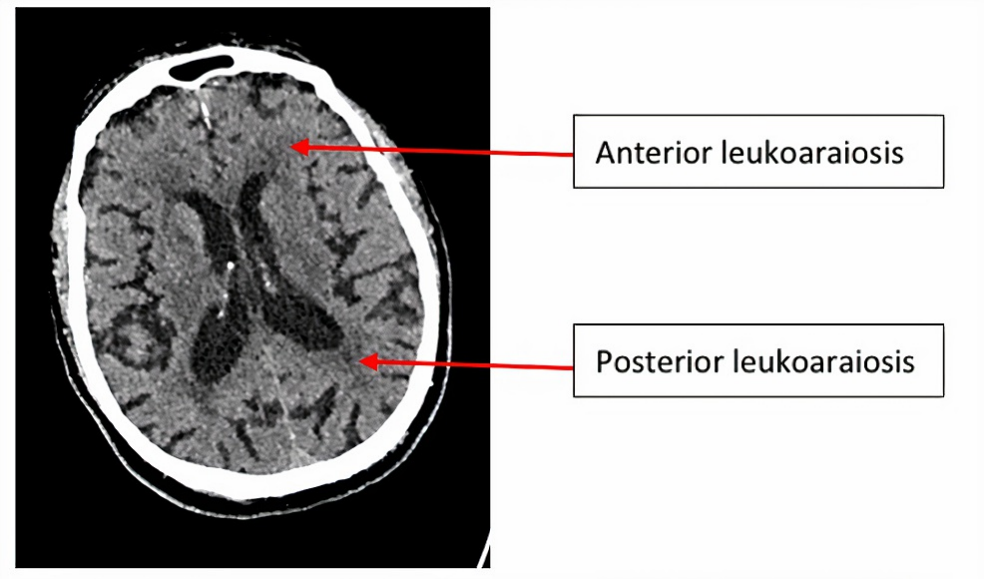
Axial CT of the brain showing anterior and posterior leukoaraiosis

CT brain imaging was assessed based on previous reports [10] for the presence of atrophy, leukoaraiosis, and old vascular lesions. Atrophy was assessed separately in cortical and central regions, defined as 0 = absent, 1 = moderate, or 2 = severe, and compared against a standard template (Figure 4) [11], thus providing a maximum score of 4. Leukoaraiosis was assessed separately in anterior and posterior brain regions [12], defined as 0 = no lucency, 1 = lucency restricted to region adjoining ventricles, or 2 = lucency covering the entire region from lateral ventricle to cortex (Figure 5), providing a maximum score of 4. Old vascular lesions/infarcts were classified by location (e.g., cortical, striatocapsular, border zone, lacunar).

**Figure 6:**
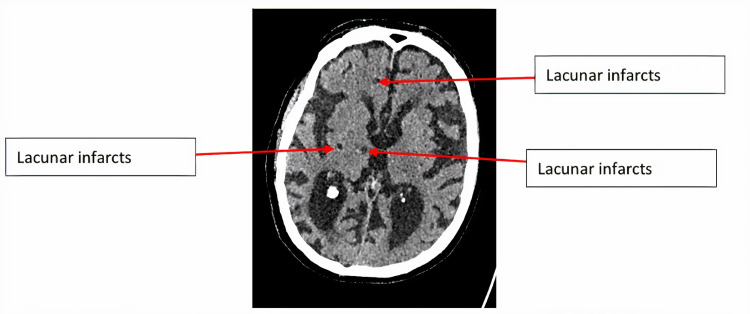
Axial CT of the brain showing lacunar infarcts

In addition to individual imaging markers of small vessel disease (SVD), we applied scores adapted for CT scanning as follows: SVD score comprises 1 point each for severe leukoaraiosis (score = 2 anteriorly and/or posteriorly as above), severe atrophy (score = 2 cortically and/or centrally), and any old lacunar infarcts/lacunas (Figure 6; maximum 3 of 3) [13]. Brain frailty score comprises 1 point each for leukoaraiosis (score = 1 or 2 anteriorly and/or posteriorly), cerebral atrophy (score = 1 or 2 cortically and/or centrally), and old vascular lesions/infarcts (maximum 3 of 3).

Statistical analysis

Data were analysed using Stata/SE 16.1 (StataCorp LLC, TX, USA). Descriptive data were displayed as frequencies, mean (SD), and median (IQR). A correlation matrix was derived utilising Pearson’s correlations for continuous variables and Spearman’s correlations for ordinal variables (CFS, brain frailty) to assess associations between age, CFS, brain frailty score, muscle quantity measurements at both C3 and L3, and adipose tissue at L3. Statistical significance was set at p<0.05.

## Results

We included 111 trauma patients. The median duration of stay in the hospital was six days (IQR 1 - 23), with a total of 17 deaths during the admission or within one year of the event. Excluding patients who were discharged to rehabilitation/step-down units, or hospices, three patients (4%) required increased levels of care on discharge. Full patient demographics and outcome data are displayed in Table 1.

**Table 1:** Demographics of patients included within the study

		Overall (total patients=111)
Demographics		
Age – mean (SD)		49.0 (25.6)
Gender – %females (N)		65.8 (73)
Ethnicity – % (N)	White	60.5 (69)
	South Asian	14.0 (16)
	Black	0.9 (1)
	Mixed or other	3.5 (4)
	Missing	21.1 (24)
Previous residence	Own home	86.0 (98)
	Own home with care	5.3 (6)
	Residential care home	2.6 (3)
	Nursing home	3.5 (4)
	Missing	2.6 (3)
CT findings		
Traumatic brain injury – % (N)		19.8 (22)
Spinal injury – % (N)		29.0 (33)
Upper limb fractures – % (N)		23.7 (27)
Rib fractures – % (N)		34.2 (39)
Pelvic fractures – % (N)		11.4 (13)
Lower limb fractures – % (N)		18.4 (21)
Other major injuries – % (N)		23.7 (27)
Outcomes		
Length of stay – median (IQR)		6 (1 – 23)
Increased care on discharge – % (N)		4.0 (3)
Inpatient death – % (N)		9.0 (10)
One year mortality – % (N)		15.3 (17)

Association of brain frailty score with age and CFS

**Figure 7:**
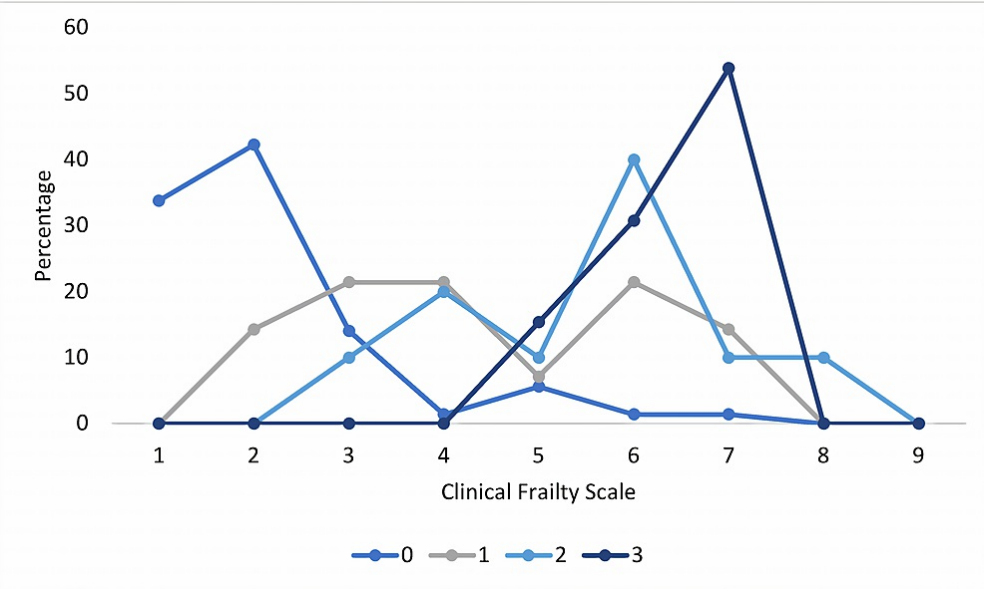
Relationship between brain frailty score and Clinical Frailty Scale (X-axis)

Brain frailty score strongly positively correlated with age (r=0.791, p<0.001), and CFS (r=0.760, p<0.001). Figure 7 shows the relationship of brain frailty with CFS as histograms for each brain frailty score; higher CFS were seen with higher brain frailty scores. Each line (Figure 7) represents each grade of brain frailty from 0 to 3.

Association of different muscle quantity measurements at L3 and C3

**Figure 8:**
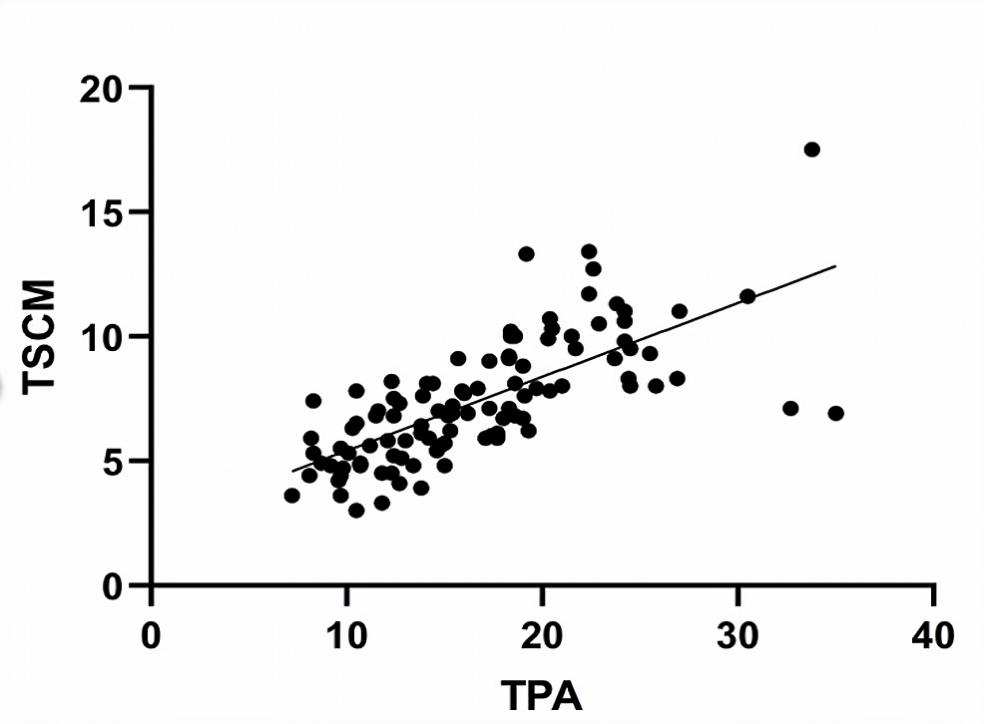
Relationship between sternocleidomastoid muscle at C3 and total psoas area at L3

At L3, TPA strongly correlated with SMM-L3 (r=0.800, p<0.001). Adipose-L3 did not correlate with SMM-L3. At C3, SMM-C3 strongly correlated with both SCM (r=0.814, p<0.001), and PVM (r=0.814, p<0.001). SMM-C3 also correlated with SMM-L3 (r=0.746, p<0.001), as did other muscle measurements (TPA and SCM: r=0.711, p<0.001; Figure 8).

Associations between age, CFS, brain frailty score, and muscle quantity at C3 and L3

**Figure 9:**
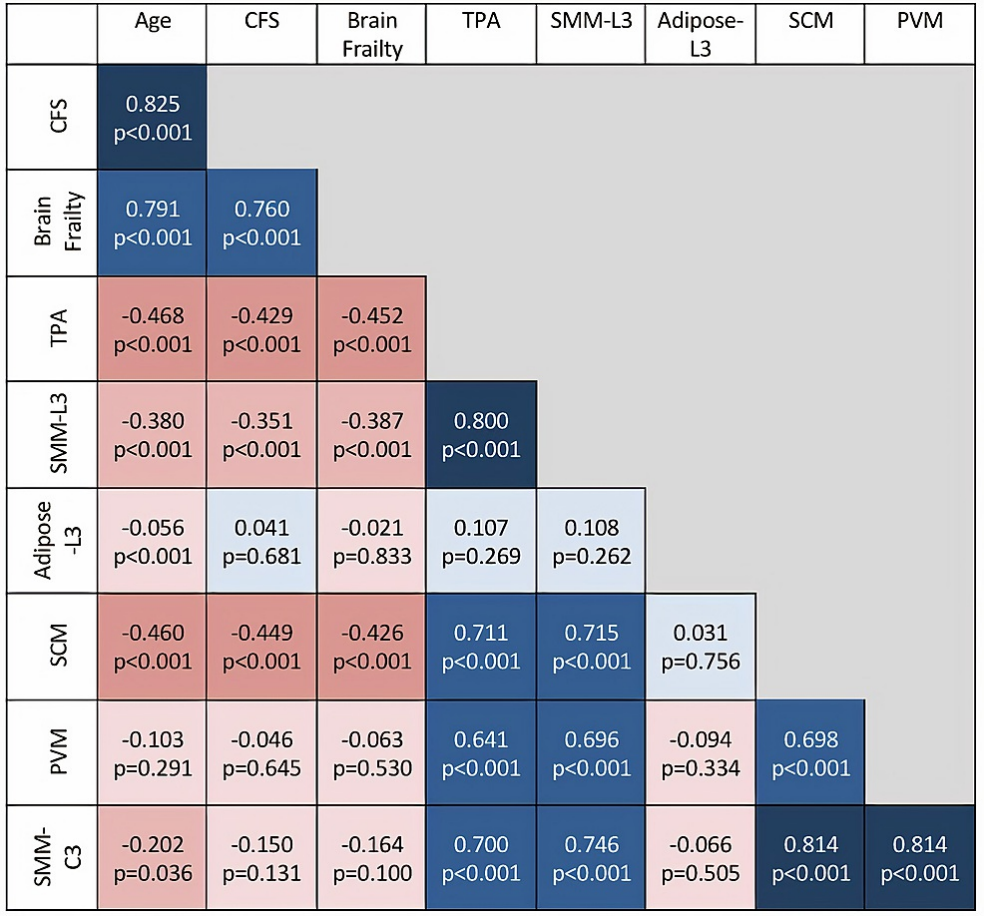
Correlation matrix of correlations between muscle quantity measurements, age, Clinical Frailty Scale, and brain frailty score Blue squares represent positive correlations and orange squares represent negative correlations. Darker squares represent stronger correlations, with lighter squares representing weaker correlations.

There were moderate negative correlations between muscle quantity measurements and age, CFS, and brain frailty. These associations were most consistent with TPA at L3 (age r=−0.468, p<0.001; CFS −0.429, p<0.001; brain frailty r=−0.452, p<0.001), and SCM at C3 (age r=−0.460, p<0.001; CFS −0.449, p<0.001; brain frailty −0.426, p<0.001; Figure 9).

## Discussion

Interpretation of results 

TPA closely correlates with SMM-L3, and all the neck CSA measures closely correlate. At L3, TPA correlates slightly better with age and CFS than SMM-L3. At C3, SCM correlates best with age and CFS. Brain frailty score correlates strongly with age and CFS, and brain frailty score similarly correlates with muscle quantity measures. Adipose in itself has no relationship between age, CFS, or any of the muscle readings. Most of the results were as expected; the most clinically significant finding was the relationship between CFS and brain frailty. These results support existing theories of the association of brain pathology with the progression of physical frailty in older adults [13,14].

How do these results relate to the current literature?

The results are in line with previous research which demonstrated that muscle quantity at C3 correlated with muscle quantity at L3 in head and neck cancer patients [7]. Previous research has not assessed the relationship between brain frailty score and physical frailty, muscle quantity, or sarcopenia. However, brain frailty score has been shown to predict cognitive and functional outcomes in patients following acute stroke disease [10]. Frailty is a known mediator of adverse outcomes in geriatric patients [15,16]

Limitations of the study 

For this study, we have taken data from only a single centre, and this may not be representative of patients presenting at other hospitals. The study is also retrospective, and mainly reliant on documentation from the emergency department. A relatively small sample size of elderly patients, for the total number of patients, was assessed as a part of the study. Due to sample size, we were also unable to look at between-group differences for gender or ethnicity. All muscle mass scores were assessed by the same assessor; as a result, were not blinded, but it is likely that these scores have less inter-rater variability. The brain imaging scores were determined by two independent assessors.

Recommendations for clinical practice

Although magnetic resonance imaging (MRI) is considered the gold standard method for determining skeletal muscle volume [17-19], a single slice CT image of L3 or C3 is more easily available. This can be used for the delineation and calculation of TPA and/or SCM quickly and easily with minimal training and may offer additional information as part of a holistic assessment. Identification of patients with low muscle quantity may be used as adjuncts to enable targeted dietetics/physiotherapy input, setting realistic rehabilitation goals, and prognostication.

Recommendations for future research 

TPA and SCM were the quickest and simplest to do and the associations show that these would be appropriate simple surrogate markers for pragmatic assessment of muscle quantity. Muscle quantity at C3 can potentially be used in place of L3 when only head/neck imaging is available. To better understand the implication of these results further research should aim to explore shared mechanisms in brain frailty, sarcopenia, and physical frailty, and better understand these relationships. Identifying these shared mechanisms may enable the development of targeted interventions towards all conditions.

## Conclusions

Muscle quantity was associated with both CFS and radiological brain frailty score. The methods used in this study may provide clinicians with an additional screening tool to assess for patient vulnerability at ease in patients, particularly older adults, without additional burden for the patient. This will enable targeted interventions including physiotherapy and dietetics input, and enable a more holistic focus of care for these patients.
